# Regional health care profiles – an improved method for generating case studies on the catchment areas of envisaged primary health care units in Austria: a report to the InfAct Joint Action

**DOI:** 10.1186/s13690-022-00821-6

**Published:** 2022-02-14

**Authors:** Stefan Mathis-Edenhofer, Florian Röthlin, David Wachabauer, Romana Haneef, Ilana Ventura, Gerhard Fülöp

**Affiliations:** 1 The Austrian National Public Health Institute (Gesundheit Österreich GmbH, GÖG), Stubenring 6, 1010 Vienna, Austria; 2 Department of Non-Communicable Diseases and Injuries, Santé Publique France, 12 rue du Val d’Osne, 94415 Saint-Maurice, France; 3 Federal Ministry of Social Affairs, Health, Care and Consumer Protection, Vienna, Austria

**Keywords:** Primary health care, Needs assessment, Catchment area, Health care service supply, Health status indicators, Health facility planning

## Abstract

**Background:**

The recent Austrian Primary Care Act established new primary health care units (PHCUs) and obliged them to draw up a “care strategy” specifying their focal care tasks and objectives and emphasizing the health care needs of the population in their catchment area with its specific local health and epidemiological profile. The main purpose of these care strategies is thus to ensure that care-providers meet the local needs, but they also provide a rationale for evaluation and organizational development. To assist new PHCUs in establishing care strategies it was necessary to develop a method for automatically generating comprehensive local case studies for any freely definable location in Austria.

**Results:**

We designed an interactive report generator capable of producing location-specific regional health care profiles for a PHCU located in any of Austria’s 2122 municipalities and of calculating the radius of its catchment area (defined by different levels of maximum car-travelling times). The reports so generated, called “regional health care profiles for primary health care” (RHCPs/PHC), are in comprehensive PDF report format. The core of each report is a set of 35 indicators, classified under five health and health service domains. The reports include an introductory text, definitions, a map, a graphic and tabular presentation of all indicator values, including information on local, supra-regional and national value distribution, a ranking, and numbers of service providers (e.g. pharmacies, surgeries, nursing homes) located within the catchment area.

**Conclusions:**

The RHCPs/PHC support primary health care planning, efforts to improve care-effectiveness, and strategic organizational development by providing comprehensive information on the health of the population, the utilization of health services and the health care structures within the catchment area. In addition to revealing the scope and nature of the health care needed, they also provide information on what public health approaches are necessary. RHCPs/PHC for different locations have already been distributed to numerous stakeholders and primary health care providers in Austria.

## Background

The Austrian Primary Care Act [1] is the result of a major health policy effort to ensure a continued high level of quality in Austria’s solidarity-based health system. The cornerstone of the Act is the legal definition of primary health care units (PHCUs), a novel organizational vehicle for the provision of primary health care. To ensure that PHCUs meet local care needs, they are required to provide health care close to the patient’s home, with needs-based opening hours and access for acute cases outside these hours. The care to be offered by PHCUs covers a broad spectrum of diagnostic, therapeutic and nursing services with several additional tasks. One specific goal, if appropriate and practical from a medical point of view, is for PHCUs to provide acute treatments and consequently reduce the load on specialist care. However, several other tasks, ranging from care for people with chronic illnesses to health promotion and disease prevention, are also within their remit.

Adopting a comprehensive inter-sectoral and multi-disciplinary approach, these new PHCUs represent a break with the traditional structure of the Austrian primary health care landscape, which has largely been based around the practices of ambulatory physicians. In PHCUs, general practitioners and other employees with backgrounds in different health and social professions collaborate to provide effective care. A PHCU can be organised centrally or in the form of a network.

Each individual PHCU is obliged to create a specific care strategy that identifies local care tasks and objectives for the unique local health and epidemiological profile of the population in its catchment area. This plan must contain information on opening hours, service provision, the structure and competences of the unit’s care team, and modes of coordination and cooperation with other health care providers. The strategy thus provides a basis for evaluation as well as a rationale for strategic organisational-development processes and is accordingly a central document in tendering processes for contracts with Austrian social security and statutory health insurance organisations.

One of the ways in which we assisted PHCUs in drawing up their care strategies was to develop a method for automatically generating comprehensive RHCPs/PHC, which provide a complete description of any given municipality chosen as the location of a PHCU together with its surrounding catchment area. The approach adopted was inspired by InfAct [2] with its many examples from other European countries of the innovative use of data sources (Deliverable WP 9.2, Part B). The regional health care profiles include five domains of health information about the catchment area: 1. Demography and socio-economic status, 2. Disease prevention and risk factors, 3. Epidemiology and mortality, 4. Health care service supply, 5. Outpatient care utilisation.

## Methods

The automatic generation of comprehensive RHCPs/PHC was developed in steps. First, we designed the core structure of the RHCP/PHC report, which comprises 35 indicators covering five health care and health service domains (Table 1) selected in a process involving feedback from physicians and other health care providers. The selection was based on five criteria: (1) legal requirements (e.g. care mandates laid down in the Primary Care Act); (2) availability of regional data (data sources are representative and valid down to the level of “health service regions” (*Versorgungsregionen*), an Austrian territorial unit level between counties and NUTS-3 regions); (3) the relative stability over time of both indicator magnitude and regional rank, even in a small-scale regional analysis; (4) relevance for the tasks of PHCUs; (5) relevance in the eyes of physicians and other care providers in PHCUs.

**Table 1:** List of indicators (*N* = 35) used in the RHCPs/PHC

**Demography, Socio-Economics**	
1. Population of the catchment area	
2. Proportion of children under 14 years (<15a)	
3. Percentage of population aged 65 and over	
4. Percentage of population aged 75 and over	
5. Percentage of population aged 65 and over in one-person households	
6. Average income per income-receiver	
**Disease Prevention and Risk Factors**	
1. Percentage with “very bad” or “bad” self-reported health, inhabitants ≥15a	
2. Percentage of persons who smoke daily or occasionally, inhabitants > = 15a	
3. Percentage of population ≥ 15a with too little exercise	
4. Percentage of population ≥ 15a with obesity	
**Epidemiology and Mortality**	
1. Life expectancy at birth (men)	
2. Life expectancy at birth (women)	
3. Prevalence of diabetes mellitus Type 2	
4. Prevalence of mental disorders	
5. Prevalence of disorders of the musculoskeletal system	
6. Prevalence of chronic head-, neck- and backaches in population ≥ 15a	
7. Percentage of long-term care benefit recipients / level 1–3	
8. Percentage of long-term care benefit recipients / level 4–7	
9. Rate of inpatients with heart disease within 2 years	
10. Rate of inpatients aged 65 and above with femoral neck fracture within 2 years	
11. Rate of inpatients with cerebrovascular disease within 2 years	
12. Rate of inpatients with cancer within 2 years	
**Health Care Service Supply**	
1. Inhabitants per general practitioner	
2. Percentage of statutory health insurance general practitioners aged 55 +	
3. Inhabitants per private general practitioner	
4. Children per paediatrician	
5. Inhabitants per statutory health insurance internist	
6. Distance to the nearest acute care hospital (incl. branch hospitals), minutes by car	
7. Number of pharmacies in the catchment area (excluding hospital pharmacies)	
8. Distance to the nearest nursing home (minutes by car)	
9. Inhabitants aged 65 and over per retirement home or nursing home in the catchment area	
**Outpatient Care Utilisation**	
1. Percentage of population visiting statutory health insurance general practitioners	
2. General practitioner equivalents per 100,000 population (incl. hospital outpatient departments)	
3. Paediatrician equivalents per 100,000 children (<15a; incl. hospital outpatient departments)	
4. Internist equivalents per 100,000 population (incl. hospital outpatient departments)	

**Fig. 1:**
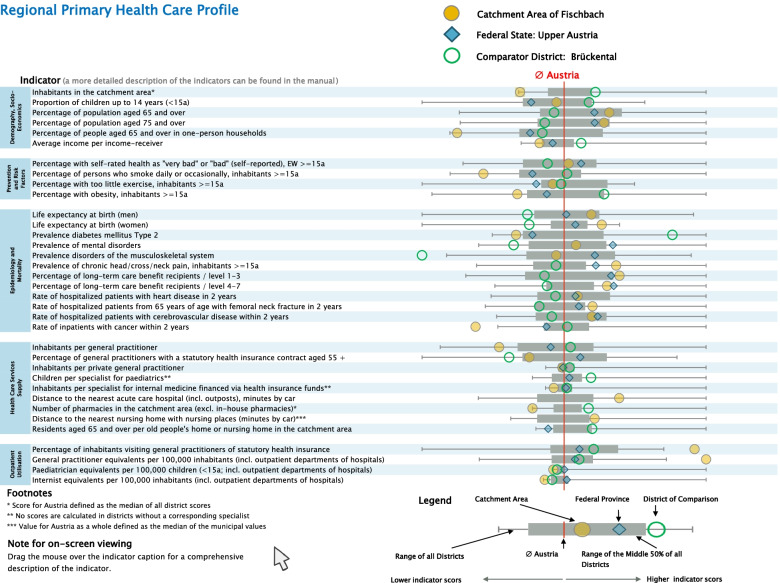
Page 2 of the RHCP/PHC: visualization of the 35 indicators from the RHCPs/PHC (using fictive data for demonstration purposes)

The second step was to define how the information provided by the indicators is presented. Figure 1 shows the visualisation design used in the RHCPs/PCH.

Our visualisation technique is based on the design used in the EU project “I2SARE” (see [3], for example). Currently, the I2SARE presentation technique is routinely used by several EU Member States but has been redesigned in more detail for our report generator. What is new in our approach is, firstly, the freedom it offers to select a municipality for a PHCU anywhere in the country and, secondly, the strategy of using catchment areas that are not bound by district (*Bezirk*) borders and therefore reflect the expected clientele of the PHCU. Additionally, unlike the representation of distributions common in scientific contexts, we used more intuitive aggregate measures: the total range, the 25 and 75 percentage quantiles and the median to depict the indicator value distribution over Austria’s districts. The visualization of 35 indicators is grouped under five domains and is implemented by box plots and overlaid circles/squares (Fig. 1).

Step one (selection of indicators) and step two (design of the output report) were accompanied by a series of feedback rounds with primary health care providers and experts in the field of primary health care with the aim of optimising the indicator set and making its visualisation intuitively understandable. The application test was carried out together with physicians and non-physician primary health care experts in the initial phase of the project. In order to address new requirements for the RHCPs/PHC (as these can change dynamically), an annual update based on user feedback is foreseen.

The third step was the implementation of an interactive report generator to produce location-specific RHCPs/PHC. The report generator is based on the data and functionalities of an integrative geographical information system already available in the Austrian Health Information System (ÖGIS) [4] but is implemented as a stand-alone application. ÖGIS provides ready-prepared, quality-assured and regionally integrated data sources that can be fed into the report generator after pre-processing. For some indicators new external data sources had to be integrated into the system and linked to those already existing. To provide the report generator with appropriate input the functionalities of ÖGIS had to be extended with SQL-bots generating datapoints for indicators for all possible location municipalities for predefined car travel-time isochrones (=catchment areas). This allows an automated output of the approximately 6500 datapoints needed as input for each indicator in one single output process. In order to concentrate on the indicators’ key data, a function for the automatic cleaning of outliers has also been implemented. Regional health care profiles combine data at a high level of spatial resolution (i.e. a total of 2122 Austrian municipalities) and comprise 35 indicators. Indicators for each of the 2122 municipalities are calculated as averages of all municipalities within a 10-, 15- and 20-min car travel-time isochrone of the PHCU location municipality. We used mainly SQL scripting to pre-process the large amounts of data and a Microsoft Excel file as the automatic report generator. Record linkage at the level of anonymised individual personal identifiers played a role for four indicators (Rate of inpatients with heart disease within 2 years, Rate of inpatients aged 65 and above with femoral neck fracture within 2 years, Rate of inpatients with cerebrovascular disease within 2 years, and Rate of inpatients with cancer within 2 years).

To generate a report the user must specify a freely selectable location municipality and a maximum car-travel time to define the radius of its catchment area. The report thus generated is a RHCP/PHC – in the format of a comprehensive PDF report.

## Results

**Fig. 2:**
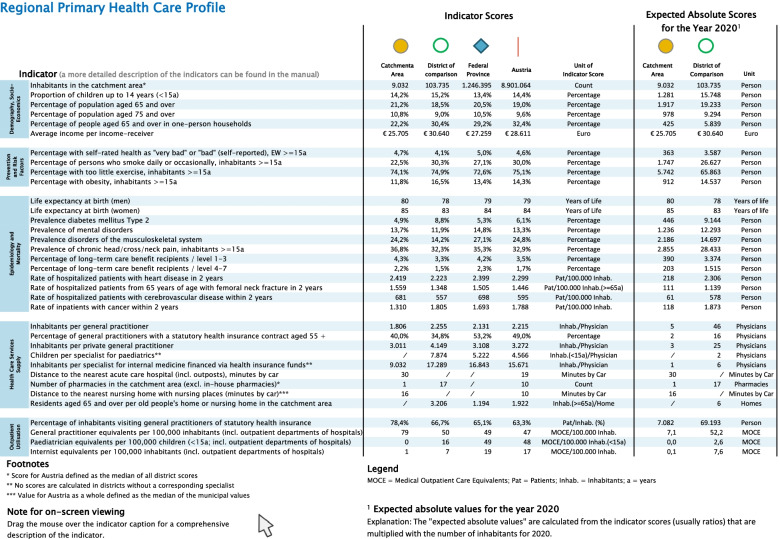
Page 3 of the RHCP/PHC: indicator results and absolute values for the catchment area in tabular form (using fictive data for demonstration purposes)

The output report - the RHCP/PHC - includes an introductory text, a graphic representation of all indicator values (Fig. 1), the indicator results and absolute values relating to the catchment area shown in tabular form (Fig. 2). This makes it possible to estimate the size of the catchment area’s population.

**Fig. 3:**
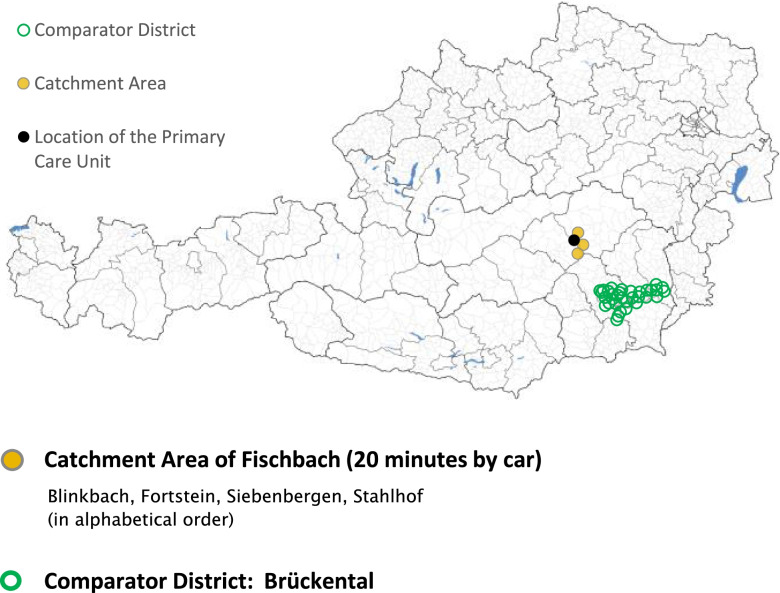
Page 1 of the RHCP/PHC: map showing the location selected for the envisaged PHCU, its catchment area and comparator district (using fictive data for demonstration purposes)

Additionally, the report includes a detailed description of each indicator (pages 4 and 5 of the RHCP/PHC are indicator definitions not shown here). Finally, the indicator values and the location (black dot) and the catchment area (ochre-coloured) of the PHCU are displayed on a map (Fig. 3).

Optionally, a comparator district (green) can be selected and used for a head-to-head comparison. Austria has 116 political districts (*Bezirke*), which are regional units well-suited for comparison with catchment areas because their populations are of a comparable order of magnitude. The final output report - the regional health care profile - is a printable PDF file. When viewed on-screen, interactive overlays are available on selected titles to call up additional information.

Interpretation of the RHCP/PHC: Each report on one of the 2122 available locations comprises a case study giving an overview of health-care-related characteristics within the catchment area. The local RHCP/PHC report is intended as a basis for the design of the PHCU. Below, some of the characteristics which can be gleaned from the indicators are considered hypothetically, and we give examples of how they could be interpreted and their potential implications for the design of a PHCU.

### Demography, socio-economics

If the age distribution of the population reveals a high proportion of elderly inhabitants in the catchment area, this could indicate that more services catering for older people need to be planned for the future PHCU. This is an example of how the indicators can be interpreted to identify target groups. The ‘average income per income-receiver’ represents the income situation of the regional population. A low value for this indicator might suggest that efforts need to be made to improve accessibility.

### Disease prevention and risk factors

The proportion of the population in a self-assessed ‘(very) poor’ state of health indicates the need for assistance in coping with everyday tasks. If this is high, the PHCU may need to offer special outreach services. The health behaviour indicators include the share of the inhabitants who smoke, do not exercise enough or are obese. These determinants indicate that more disease-preventing and health-promoting measures should be implemented.

### Epidemiology and mortality

Life expectancy at birth is often linked to limiting structural factors. The prevalence of diabetes mellitus type 2, mental disorders, disorders of the musculoskeletal system or chronic head-, neck- and backaches indicate the occurrence of typical diseases in the catchment area which might require targeted managed-care programs. The proportion of people living in single-person households aged 65+ points to those living alone who might be suffering from limited social participation or in need of greater support. The share of long-term care allowance recipients and the proximity and number of nursing homes in the catchment area indicate a potential increase in the need for outreach services.

### Health care services supply

The presence of existing services in the fields of general medicine and paediatrics or in other health care structures (hospitals, pharmacies, nursing facilities) can answer questions about the necessary structure and sizing of the personnel pool within the envisaged PHCU and point out possible cooperation within the region-specific primary care tasks.

### Outpatient care utilisation

There are often regional differences in the utilisation of health services. Below-average values for these indicators (average values are displayed as a red line in the figure on page 2 in Appendix 2) may indicate access problems or a ‘relatively healthy population’. Above-average values may indicate special regional care needs that could be covered by the envisaged PHCU.

The interpretation of the local RHCP/PHC is carried out by the entities applying to establish a PHCU. To assist initiators of PHCUs with the interpretation of the local RHCP/PHC report we additionally provide a manual for users. This describes in more detail how regional profiles can be used to develop a primary care strategy and includes a comprehensive example of how the indicators’ results can be interpreted to this end.

## Discussion

In this paper we present a method for generating location-specific regional health care profiles for primary health care based on indicator sets. This method is partially an improvement of one used in the EU project I2SARE. To our knowledge, Austria is the first country in Europe that can rely on a method to automatically generate RHCPs/PHC at such a detailed level of spatial resolution (i.e. for a total of 2122 Austrian municipalities) and to the extent of 35 related indicators. The report generator provides targeted decision support for new PHCUs in any given municipality in Austria. The output is a comprehensive profile that describes the regional health care and health service needs within the catchment area of a PHCU. The report includes an introductory text, both graphic and tabular representation of all indicators, a map of the location municipality and its catchment area and a detailed description of each indicator. The report is also accompanied by a user manual with a guide to creating a care strategy for a PHCU.

The RHCP/PHC is an example of innovative use of health care data and cross-domain data linkage. The data needed to calculate the indicators are derived from more than 10 different sources: population data, survey data, routine inpatient and outpatient data, income statistics, long-term care allowance data, life expectancy data, statistics on health professionals, health care utilisation data and geographical data. All these data sources are queried regularly to provide up-to-date indicator values.

The RHCP/PHC delivers a clearly identifiable benefit for the health system. Local versions of the RHCP/PHC have already been distributed to numerous institutional stakeholders and care providers in Austria to assist in defining the services to be offered by individual PHCUs. By disseminating the regional health care profiles, we can provide locally targeted information to support health care professionals in the process of establishing new PHCUs.

There are, however, some important limitations. The first of these concerns the data sources. As certain data were not available at the municipality level, calculations for 8 indicators (the data sources affected were mostly survey data) had to be interpolated from higher regional levels. This leads to a blurring of regional outputs in each of the 2122 municipalities. Secondly*,* the size of the catchment areas and their populations is based on accessibility by car, which varies greatly according to the degree of urbanization, and thus limits the comparability of different catchment areas. Thirdly, due to restricted usage rights for certain data sources, health care profiles cannot be disseminated freely and an approval process needs to be followed. Fourthly*,* the set of indicators is limited due to a lack of regional data in some instances (e.g. physiotherapists, occupational therapists and speech therapists). Finally, data on accessibility by public transport has not yet been implemented.

## Conclusion

RHCPs/PHC play an important role in determining and describing the aims of envisaged primary health care institutions by providing comprehensive information on population health, utilization of health services and health care structures. In addition to assessing the scope and nature of health care, they also provide information on the public health approaches that are needed. Regional health care profiles are an example of innovative use of health information and information technologies.

## Data Availability

Details of the report generator and the algorithms implemented can be obtained from the corresponding author. Local versions of the regional health care profiles for primary health care are available exclusively to those applying to establish primary health care units in Austria.

## Abbreviations

a: Years
EU: European Union
InfAct: Information for Action, a joint project of Member States to establish a sustainable European health information system
I2SARE: Health Inequalities Indicators in the Regions of Europe
PHCU: Primary health care unit
RHCP/PHC: Regional health care profiles for primary health care
WP: Work package

## References

[1] Federal law: Primary Care Act (2021). https://www.ris.bka.gv.at/GeltendeFassung.wxe?Abfrage=Bundesnormen&Gesetzesnummer=20009948. Accessed 27 May 2021.

[2] European Union: Joint Action on Health Information (2018). https://www.inf-act.eu. Accessed 5 May 2021.

[3] Health Inequalities Indicators in the Regions of Europe (I2SARE): Regional Health Profiles in the European Union – Spain, Galicia. https://www.sergas.es/Saude-publica/Documents/2482/I2SARE%20Perfil%20de%20Galicia.pdf. Accessed 22 Nov 2020.

[4] The Austrian National Public Health Institute (Gesundheit Österreich GmbH, GÖG): Austrian Health Information System (2018). https://goeg.at/OEGIS. Accessed 7 Jan 2021.

